# Association between diabetes mellitus and trochanteric bone mineral density in individuals with osteoporotic fractures: a retrospective study

**DOI:** 10.3389/fmed.2024.1492603

**Published:** 2024-12-17

**Authors:** Shao-han Guo, Jian Xu, Min-zhe Xu, Chong Li, Ya-qin Gong, Ke Lu

**Affiliations:** ^1^Department of Orthopedics, Affiliated Kunshan Hospital of Jiangsu University, Suzhou, Jiangsu, China; ^2^Department of Orthopedics, The First People’s Hospital of Kunshan, Gusu School, Nanjing Medical University, Suzhou, Jiangsu, China; ^3^Information Department, Affiliated Kunshan Hospital of Jiangsu University, Suzhou, Jiangsu, China

**Keywords:** osteoporotic fractures, diabetes, bone mineral density, sex-specific effects, retrospective analysis

## Abstract

**Background:**

The relationship between diabetes mellitus (DM) and bone mineral density (BMD) in patients with osteoporotic fractures (OPFs) remains complex and heterogeneous, specifically between the genders. This study aimed to explore the association between diabetes status and trochanteric BMD in a cohort of patients with OPFs and elucidate the differences between male and female patients.

**Methods:**

This retrospective analysis was performed on 710 admitted patients aged 50 years or older with OPFs. In this study, the exposure variable was diabetes status. Trochanteric BMD comprised the dependent variable. While controlling for covariance influences, generalized estimating equations (GEE) were applied to examine the independent link between diabetes status and trochanteric BMD in OPFs patients. Moreover, a subgroup analysis was also conducted to validate the result’s stability.

**Results:**

A substantial positive association was noted between diabetes status and trochanteric BMD in diabetic patients, as determined by the fully adjusted model (*β* = 0.017, 95% CI 0.001 to 0.033, *p* = 0.035). Furthermore, the sex-specific analysis showed a significant positive relationship between diabetes status and trochanteric BMD in male patients (*β* = 0.040, 95% CI 0.006 to 0.075, *p* = 0.022), whereas no significant relationship was observed in female patients (*β* = 0.010, 95% CI −0.008 to 0.028, *p* = 0.256).

**Conclusion:**

This study highlighted the significant sex differences in the impact of diabetes on trochanteric BMD among patients with OPFs. The male diabetic patients had higher trochanteric BMD than their non-diabetic counterparts; however, this association was not evident in female patients. Further research is necessary to understand the underlying mechanisms that contribute to these sex-specific differences and to evaluate the clinical implications of managing fracture risk in diabetic patients.

## 1 Introduction

Osteoporosis (OP) is a very common skeletal disorder, which is marked by reduced bone mass, impaired integrity of bone tissue, increased bone fragility, and a higher susceptibility to fractures (1). Osteoporosis affects about 200 million people worldwide (2), with an osteoporotic fracture occurring every three seconds globally (3). Therefore, it is a major public health concern with significant socioeconomic impact. Osteoporotic fractures (OPFs) can be diagnosed by weakened bone mineral density (BMD) and strength, which collectively contribute to an elevated vulnerability to fractures. BMD measurement is often used in the evaluation of fracture risk; it is widely recognized as a crucial indicator of skeletal health (4). BMD is a biomarker of considerable importance in assessing fracture risk and bone quality. It quantifies the amount of inorganic salts within a specific mass of bone tissue (5). In the geriatric population, OPFs are a substantial factor in morbidity and mortality. Among all osteoporotic fractures, hip fracture is one of the most serious complications of aging, and it is the 7th leading cause of death in older adults (6). The incidence of hip fractures is increasing, primarily due to the demographic transition occurring on a global scale, resulting in an extended life expectancy. A substantial proportion, approximately 50%, of all hip fractures are trochanteric fractures. The average annual cost per patient due to trochanteric fractures is estimated to exceed $50,000 (7). Moreover, it is expected that the incidence of trochanteric fractures relative to cervical fractures will progress at a more accelerated rate (8). This demonstrates the growing relevance of treating trochanteric fractures in the discipline of orthopedics. Given these considerations, orthopedic specialists should pay particular attention to such fractures and develop more effective treatment strategies.

Diabetes mellitus (DM) is a prevalent medical condition that manifests globally. Based on epidemiological data, the global prevalence of diabetes among the 18–99 age group was estimated to be around 451 million people in 2017. This number is predicted to undergo a substantial increase, resulting in around 693 million by 2045 (9). In addition to damage to patients’ bodies and minds, diabetes and its variegated complications also impose a heavy financial burden on families and society. OPFs have emerged as a significant complication of diabetes (10). Accumulating evidence supports a strong connection between diabetes and a higher susceptibility to OPFs (11, 12). Given these concerning trends, the study’s results highlight the critical importance of implementing effective preventive and management strategies to reduce the burden caused by this disease.

In recent years, several studies have highlighted the complex interplay between diabetes and osteoporosis. Despite higher or unchanged BMD, an increased overall risk of fractures has been observed in patients with type 2 diabetes mellitus (T2DM) (13). A review conducted by Romero-Díaz et al. (14) indicated that T2DM patients had normal or elevated BMD, though paradoxically, alterations in bone microarchitecture increase their risk of fractures. Furthermore, research indicates that elderly T2DM patients have an elevated risk of fractures in the hip, thigh, foot, humerus, and overall (15–18). The strength of bones and their susceptibility to fracture depends not only on bone mass but also on bone quality (19). In diabetic patients, BMD does not accurately reflect fracture risk, suggesting the involvement of additional pathophysiological mechanisms. Impaired bone quality may lead to increased bone fragility, thereby increasing the fracture risk, independently of BMD (20). This study retrospectively analyzed hospitalized elderly patients with osteoporosis to evaluate the potential relationship between diabetes status and trochanteric BMD among OPF patients and elucidate the association of this relationship in males and females.

## 2 Materials and methods

### 2.1 Study design and participants

**Figure 1:**
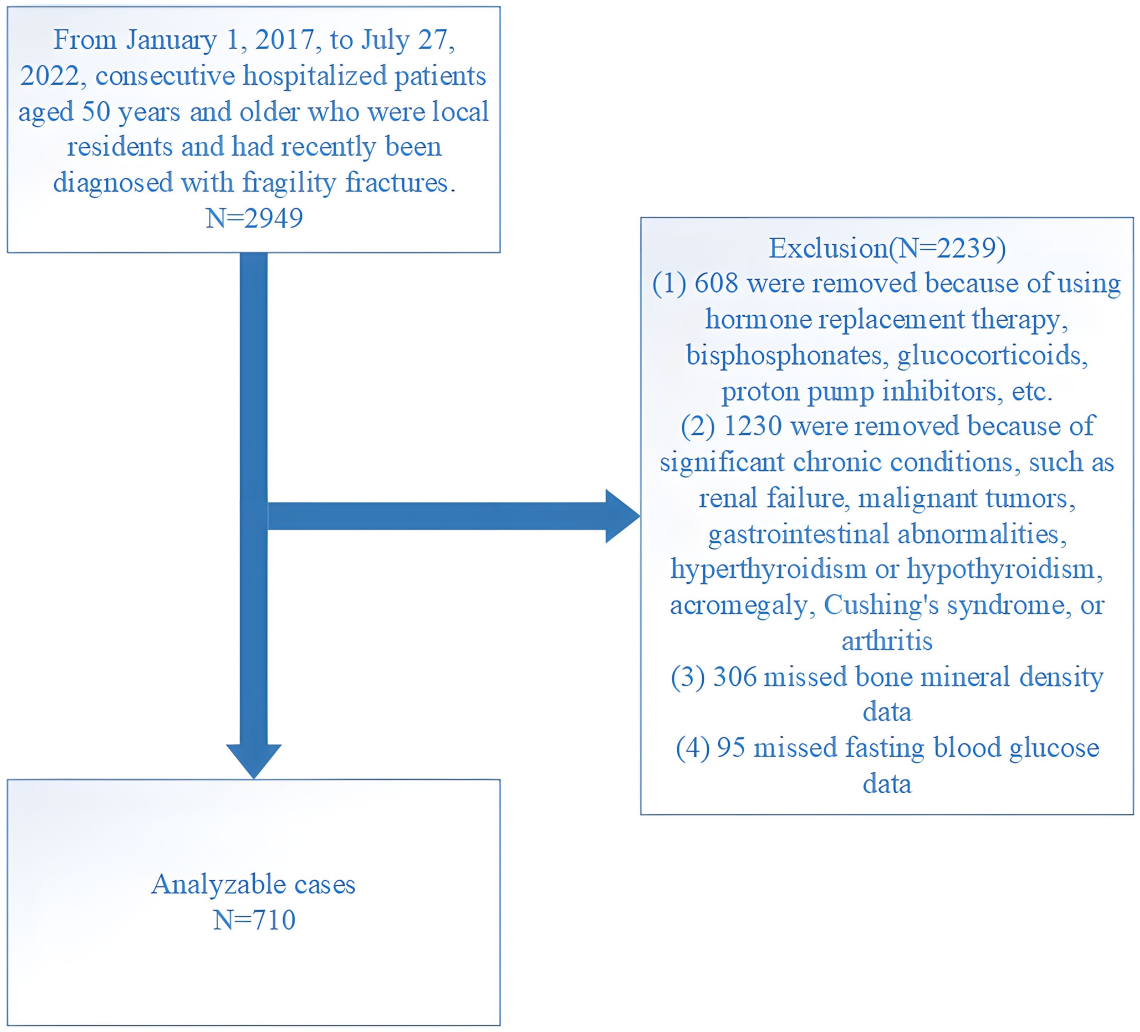
Study flowchart.

This retrospective study was conducted at the Affiliated Kunshan Hospital of Jiangsu University (AKHJU). Electronic patient records were collected from all participants aged 50 years or older who were recently admitted with the diagnosis of OPFs from January 1, 2017, to July 27, 2022. Additionally, these patients had not encountered any fractures for at least 5 years prior, marking this as their inaugural occurrence of an OPF. OPFs, also known as fragility fractures (21), are typically induced by low-energy mechanisms, such as falls from standing height or lower. Among these, hip fractures represent the most severe form of OPFs. Other types of low-trauma OPFs include certain distal forearm fractures, proximal humerus fractures, vertebral fractures, and pelvic fractures (22). These fractures are diagnosed using the International Statistical Classification of Diseases and Related Health Problems, 10th Revision (ICD-10), specifically under the codes beginning with S22, S32, S42, S52, or S72 (23). To maintain study homogeneity, high-energy fractures, such as those resulting from car accidents were excluded to ensure the focus remained on fractures typically associated with osteoporosis. To focus our analysis specifically on adults with diagnosed type 2 diabetes, we excluded individuals who were diagnosed before the age of 50 and who began insulin therapy within 1 year of their diagnosis (24). Further, non-local residents and patients who died within the initial month of admission were excluded from the analysis. Initially, 2,949 consecutive OPF individuals who received orthopedic surgery were included. The inclusion criteria were: (1) age ≥50 years; (2) fracture diagnosis confirmed by radiography or computed tomography, including fractures of the wrist, proximal humerus, lumbar vertebra, thoracic vertebra, femoral neck, and femoral trochanteric/subtrochanteric region; (3) surgical treatment in hospital; and (4) availability of hospital clinical data. Of these patients, 2,239 were excluded based on the following criteria: (1) use of hormone replacement therapy, glucocorticoids, bisphosphonates, proton pump inhibitors, or other similar medications (608 cases); (2) presence of significant chronic conditions including renal failure, malignant tumors, gastrointestinal abnormalities, hyperthyroidism or hypothyroidism, acromegaly, Cushing’s syndrome, or arthritis (1,230 cases); (3) absence of BMD results (306 cases); and (4) absence of fasting blood glucose (FBG) results (95 cases). The final analysis included 710 patients (Figure 1). The study received ethical approval from the AKHJU (approval number: 2021-06-015-K01), and we conducted all procedures in accordance with the Declaration of Helsinki. To ensure patient privacy, details regarding patients were hidden from the investigators. All participants provided written informed consent.

### 2.2 Exposure and outcome variables

In this study, the exposure variable was diabetes, which was defined as self-reported diabetes, use of glucose-lowering drugs, or FBG ≥7 mmol/L (25). An AutoAnalyzer (Beckman-Coulter AU 5800, United States) was utilized to determine the FBG level in venous blood after fasting for 8 h. The outcome variable was trochanteric BMD measured by dual-energy X-ray absorptiometry (DXA) via a Hologic dual-energy X-ray bone density instrument (Discovery Wi, Hologic Inc., United States). Following standardized procedures, every measurement was obtained using the same instrument and competent operator. The apparatus received routine quality control procedures daily in advance of participant examination.

### 2.3 Covariate analyses

Sex, age, body mass index (BMI), magnesium, phosphorus, sodium, calcium, hemoglobin, platelet count, albumin count, lymphocyte count, neutrophil count, monocyte count, alanine aminotransferase (ALT), aspartate aminotransferase (AST), creatinine (Cr), blood urea nitrogen (BUN), serum uric acid (SUA), glycated hemoglobin (HbA1c), fracture category, hypertension, American Society of Anesthesiologists (ASA) scores and Charlson comorbidity index (CCI) were main potential covariates analyzed in this study. All blood samples were obtained from fasting patients. The ASA classification is determined by anesthesiologists through a preoperative assessment of a patient’s health status, categorizing patients in accordance with the severity of their underlying diseases and their potential influence on anesthesia management (26). In contrast, the CCI score is calculated by allocating specific weights to comorbidities, which are determined based on their relative effect on patient mortality. Comorbidities taken into account encompass cardiovascular disease, diabetes, cancer, and renal disease, with each condition being assigned a weight ranging from 1 to 6. Higher weights signify a greater influence on mortality. The total CCI score, which is the summation of the weights for all diagnosed conditions, reflects the overall burden of comorbidities and assists researchers and clinicians in evaluating their impact on mortality and postoperative complications (27). Fracture category, which includes fracture of the lumbar vertebra, thoracic vertebra, wrist, proximal humerus, femoral neck, and femoral trochanteric/subtrochanteric region.

### 2.4 Statistical analyses

EmpowerStats^1^ and R packages^2^ were employed for all statistical analyses, with a limit of the significance of a two-sided *p*-value ≤0.05. Continuous variables are presented as means with standard deviations (SD) or medians with interquartile ranges (Q1–Q3), whereas categorical data are represented as frequencies (%). For comparison between groups, Mann–Whitney *U* tests were performed to evaluate non-normally distributed data, while independent two-tailed *t*-tests were carried out for normally distributed variables. Chi-square tests were conducted to assess differences between categorical variables. In case, the chi-squared test assumptions were not met, the Fisher exact test was performed. Furthermore, univariate analyses were performed to examine the associations between OPF patient characteristics and trochanteric BMD.

The study utilized generalized estimating equations (GEE) to investigate the independent association between diabetes status and trochanteric BMD in OPF patients while controlling the confounders. To systematically evaluate this association, three models were developed: an unadjusted Model 1, a minimally adjusted Model 2, and a fully adjusted Model 3. Initially, collinearity diagnosis was conducted using the variance inflation factor (VIF). The need for covariate adjustment was then assessed according to the following criteria: Criterion 1 involved introducing covariates to the basic model, which initially comprised only diabetes status and trochanteric BMD, or removing covariates from the full mode. The full model incorporated all potential covariates including age, sex, BMI, magnesium, sodium, phosphorus, platelet count, hemoglobin, albumin, calcium, neutrophil count, lymphocyte count, monocyte count, ALT, AST, Cr, BUN, SUA, hypertension, ASA classification, and fracture category, in addition to diabetes status and trochanteric BMD. A covariate was considered necessary for adjustment if its inclusion or exclusion resulted in a ≥10% change in the odds ratio (OR). Criterion 2 required meeting Criterion 1 or having a covariate with a *p*-value < 0.1 in the univariate model (28). For model development, Model 1 was not adjusted while Model 2 was adjusted for age and BMI. Model 3 underwent further adjustments compared to Model 2, following either Criterion 1 or Criterion 2, specifically including age, BMI, hemoglobin, neutrophil, lymphocyte, monocyte, phosphorus, and platelet. Considering the sex-specific characteristics associated with diabetes status and trochanteric BMD, analyses were conducted separately for separate groups based on gender to analyze their interactions.

To assess the stability of subgroups and potential heterogeneity, we executed subgroup analyses repeatedly while categorizing a range of covariates. The interactions and modifications within subgroups were assessed utilizing the likelihood ratio test (LRT).

## 3 Results

### 3.1 Patient characteristics

This retrospective analysis included 710 patients aged 50 years and older with osteoporotic fractures, comprising 547 females and 163 males. The average trochanteric BMD among males was 0.57 ± 0.10 g/cm^2^, which was significantly higher than the 0.50 ± 0.10 g/cm^2^ observed in females (*p* < 0.001) (Table 1). There were no significant differences in age (females 71.53 ± 9.88 years, males 70.94 ± 11.07 years, *p* = 0.514) or BMI (females 22.76 ± 3.16 kg/m^2^, males 22.32 ± 3.27 kg/m^2^, *p* = 0.121) between the sexes. Males exhibited slightly higher levels of serum magnesium and sodium, while females had higher phosphorus levels, all showing statistical significance. No significant differences were observed in platelet count, hemoglobin, and albumin levels between the sexes, and the prevalence rates of diabetes and hypertension were similar across the groups.

**Table 1:** Patient characteristics based on different sex groups.

Variables	Female	Male	*p*-value^a^	*p*-value^b^
*N*	547	163		
Trochanteric BMD, mean ± SD, g/cm^2^	0.50 ± 0.10	0.57 ± 0.10	<0.001	<0.001
Age, mean ± SD, years	71.53 ± 9.88	70.94 ± 11.07	0.514	0.499
BMI, mean ± SD, kg/m^2^	22.76 ± 3.16	22.32 ± 3.27	0.121	0.125
Magnesium, mean ± SD, mmol/L	0.88 ± 0.10	0.90 ± 0.09	0.008	<0.001
Sodium, mean ± SD, mmol/L	141.23 ± 2.87	140.64 ± 2.66	0.020	0.005
Phosphorus, mean ± SD, mmol/L	1.10 ± 0.23	1.05 ± 0.19	0.007	0.019
Platelet count, mean ± SD, ×10^9^/L	174.38 ± 62.02	165.13 ± 59.64	0.093	0.045
Hemoglobin, mean ± SD, g/L	125.59 ± 18.41	123.14 ± 19.30	0.142	0.171
Albumin, mean ± SD, g/L	39.89 ± 4.27	39.81 ± 3.79	0.827	0.656
Calcium, mean ± SD, mmol/L	2.20 ± 0.13	2.20 ± 0.11	0.912	0.908
Neutrophil count, mean ± SD, ×10^9^/L	6.36 ± 3.14	6.76 ± 3.14	0.153	0.087
Lymphocyte count, mean ± SD, ×10^9^/L	1.29 ± 0.57	1.21 ± 0.58	0.109	0.054
Monocyte count, mean ± SD, ×10^9^/L	0.48 ± 0.24	0.51 ± 0.31	0.168	0.218
ALT, mean ± SD, U/L	23.97 ± 26.94	21.47 ± 12.11	0.252	0.841
AST, mean ± SD, U/L	27.07 ± 38.56	23.15 ± 8.73	0.199	0.290
Cr, mean ± SD, μmol/L	69.94 ± 34.43	63.39 ± 24.53	0.024	<0.001
BUN, mean ± SD, mmol/L	6.02 ± 3.23	6.62 ± 2.62	0.031	<0.001
SUA, mean ± SD, μmol/L	283.82 ± 99.18	285.44 ± 94.26	0.853	0.746
HbA1c, mean ± SD, %	6.84 ± 1.11	7.41 ± 2.52	0.416	0.665
Diabetes, *N* (%)			0.953	—
No	414 (75.69%)	123 (75.46%)		
Yes	133 (24.31%)	40 (24.54%)		
Hypertension, *N* (%)			0.845	—
No	446 (81.54%)	134 (82.21%)		
Yes	101 (18.46%)	29 (17.79%)		
ASA score, *N* (%)			0.533	—
1	36 (6.58%)	13 (7.98%)		
2	371 (67.82%)	103 (63.19%)		
≥3	140 (25.59%)	47 (28.83%)		
CCI score, *N* (%)			0.582	—
0	486 (88.85%)	141 (86.50%)		
1	46 (8.41%)	18 (11.04%)		
≥2	15 (2.74%)	4 (2.45%)		
Fracture category, *N* (%)			<0.001	—
Thoracic vertebra	131 (23.95%)	18 (11.04%)		
Lumbar vertebra	188 (34.37%)	65 (39.88%)		
Wrist	19 (3.47%)	3 (1.84%)		
Proximal humerus	46 (8.41%)	5 (3.07%)		
Femoral neck	98 (17.92%)	48 (29.45%)		
Femoral trochanteric/subtrochanteric	65 (11.88%)	24 (14.72%)		

SD, standard deviation; BMD, bone mineral density; BMI, body mass index; ALT, alanine aminotransferase; AST, aspartate aminotransferase; Cr, creatinine; BUN, blood urea nitrogen; SUA, serum uric acid; HbA1c, glycated hemoglobin; ASA, American Society of Anesthesiologists; CCI, Charlson comorbidity index.

a *p-value: t-tests for continuous variables, chi-square tests for categorical variables.*

b *p-value: Kruskal Wallis rank test for continuous variables, Fisher exact for categorical variables with expects <10.*

Supplementary Table S1 describes patient characteristics categorized by diabetes status. Among the 710 patients, 537 patients had no diabetes, while 173 were diagnosed with the condition. A comparative analysis revealed no significant differences in trochanteric BMD between the non-diabetic (0.51 ± 0.10 g/cm^2^) and the diabetic groups (0.52 ± 0.10 g/cm^2^, *p* = 0.188). Age and BMI also showed no significant differences between these groups. Moreover, the diabetic group had slightly lower levels of blood magnesium and sodium, whereas platelet and hemoglobin levels were higher in the non-diabetic group. Moreover, the non-diabetic group also indicated significantly higher neutrophil and lymphocyte counts compared to the diabetic group. These baseline characteristics provide a crucial context for exploring the association between diabetes and bone density.

### 3.2 Univariate analysis of trochanteric BMD

Univariate analysis highlights significant associations between several clinical factors and trochanteric BMD (Supplementary Table S2). Furthermore, age (*β* = −0.004, *p* < 0.001) and BMI (*β* = 0.008, *p* < 0.001) showed robust associations with trochanteric BMD, with age inversely related and BMI positively related. Phosphorus levels negatively influenced trochanteric BMD (*β* = −0.056, *p* = 0.001), whereas serum uric acid also presented a slight negative correlation (*β* = −0.000, *p* = 0.004). Furthermore, higher ASA scores correlated with lower trochanteric BMD, specifically in patients with an ASA score of ≥3 (*β* = −0.077, *p* < 0.001). Lumbar vertebra fractures were associated with increased trochanteric BMD (*β* = 0.028, *p* = 0.007).

### 3.3 Evaluation of the relationship between diabetes and trochanteric BMD

The relationship between diabetes status and trochanteric BMD in various models, with sex as a variable, is displayed in Table 2. The analysis comprises three distinct models: a model with no adjusted, a model with minimally adjusted, and a model with fully adjusted. There was no substantial correlation noted between diabetes status and trochanteric BMD in either males or females in the non-adjusted model. In both sex groups, the minimally-adjusted model, which adjusted for age and BMI, failed to identify a statistically significant link between diabetes status and trochanteric BMD. Further variables were incorporated into the fully adjusted model, such as the quantity of platelets, neutrophils, lymphocytes, monocytes, phosphorus, and hemoglobin levels. There was no statistically significant correlation observed between diabetes status and trochanteric BMD in the females (*β* = 0.010, 95% CI −0.008 to 0.028, *p* = 0.256). A noteworthy positive correlation was identified between diabetes status and trochanteric BMD in males (*β* = 0.040, 95% CI 0.006 to 0.075, *p* = 0.022). This implies that males with diabetes exhibited considerably greater trochanteric BMD values relative to their non-diabetic males. The correlation between diabetes status and trochanteric BMD became highly significant when both sexes were taken into account (*β* = 0.017, 95% CI 0.001 to 0.033, *p* = 0.035).

**Table 2:** Association between diabetes status and trochanteric BMD in different models.

	Diabetes	Female	Male	Total
Non-adjusted model	No	Reference	Reference	Reference
	Yes	0.007 (−0.012, 0.025) 0.501	0.028 (−0.006, 0.063) 0.109	0.012 (−0.005, 0.028) 0.174
Minimally-adjusted model	No	Reference	Reference	Reference
	Yes	0.006 (−0.011, 0.023) 0.502	0.036 (0.004, 0.069) 0.030	0.014 (−0.001, 0.029) 0.077
Fully-adjusted model	No	Reference	Reference	Reference
	Yes	0.010 (−0.008, 0.028) 0.256	0.040 (0.006, 0.075) 0.022	0.017 (0.001, 0.033) 0.035

Data in the table: *β* (95%CI) *p*-value, outcome variable: trochanteric BMD, exposed variables: diabetes. Minimally adjusted model adjusted for age and BMI. Fully adjusted model adjusted for age, BMI, hemoglobin, neutrophil, lymphocyte, monocyte, phosphorus, and platelet.

Further analyses presented in Supplementary Tables S3, S4 explored the relationship between diabetes status and BMD at various anatomical sites, with adjustments for different covariates. Supplementary Table S3, using a fully adjusted model, demonstrated a significant positive correlation between diabetes status and lumbar spine BMD (*β* = 0.026, 95% CI: 0.002 to 0.050, *p* = 0.036). Although correlations in other skeletal regions were not consistently significant, the effect sizes indicated a uniform trend across these areas. Supplementary Table S4 assessed the impact of varying sets of covariates across three models, particularly noting in Model 3 that after adjusting for age, BMI, lymphocytes, monocytes, phosphorus, platelets, BUN, hypertension, ASA score, and CCI score, a significant association was found between diabetes and trochanteric BMD in males (*β* = 0.049, 95% CI: 0.011 to 0.087, *p* = 0.013). These findings suggest that diabetes is modestly but significantly associated with an increase in trochanteric BMD, particularly in males, when a wide range of confounding factors are considered.

### 3.4 Subgroup analysis

This study stratified all subgroups by age, sex, BMI, hemoglobin levels, quantification of neutrophils count, lymphocytes count, monocytes count, phosphorus levels, and platelets to further validate the reliability of the resultant outcomes in the fully adjusted model when potential confounding variables were represented.

These covariates were adjusted in all analyses, except for the subgroup variable. A remarkably consistent pattern is presented in Table 3, and interactions were not detected across any stratification (all *p*-values for interactions were greater than 0.05).

**Table 3:** Subgroup analyses exploring the association between diabetes status and trochanteric BMD.

Subgroup	*N*	Sex = female	*p*-value for interaction	Sex = male	*p*-value for interaction	Total	*p*-value for interaction
Age, years							
50–66	232	0.01 (−0.02, 0.04) 0.42	0.881	0.02 (−0.05, 0.08) 0.58	0.643	0.01 (−0.02, 0.04) 0.42	0.967
67–75	227	0.01 (−0.02, 0.05) 0.42		0.04 (−0.02, 0.10) 0.18		0.01 (−0.02, 0.04) 0.52	
76–97	251	0.00 (−0.03, 0.04) 0.87		0.06 (−0.00, 0.11) 0.07		0.01 (−0.02, 0.05) 0.36	
BMI, kg/m^2^							
14.02–21.22	237	0.02 (−0.02, 0.05) 0.33	0.606	0.04 (−0.01, 0.10) 0.15	0.296	0.02 (−0.01, 0.05) 0.18	0.462
21.26–24.03	235	−0.00 (−0.03, 0.03) 0.95		−0.00 (−0.05, 0.05) 0.95		0.01 (−0.02, 0.03) 0.67	
24.04–33.20	238	0.02 (−0.01, 0.05) 0.23		0.05 (−0.01, 0.12) 0.11		0.02 (−0.01, 0.05) 0.22	
Hemoglobin, g/L							
53–118.6	225	0.00 (−0.02, 0.03) 0.80	0.675	0.05 (−0.02, 0.11) 0.15	0.143	0.01 (−0.02, 0.04) 0.49	0.608
119–132	231	0.02 (−0.01, 0.06) 0.21		0.01 (−0.06, 0.08) 0.71		0.02 (−0.01, 0.05) 0.25	
133–169	253	0.01 (−0.02, 0.04) 0.42		0.10 (0.04, 0.16) <0.01		0.03 (0.00, 0.06) 0.04	
Neutrophil count, ×10^9^/L							
1.3–4.6	227	0.00 (−0.03, 0.04) 0.85	0.692	0.05 (−0.03, 0.13) 0.24	0.178	0.01 (−0.02, 0.05) 0.35	0.944
4.7–7	245	0.02 (−0.01, 0.06) 0.20		−0.01 (−0.08, 0.06) 0.78		0.01 (−0.02, 0.05) 0.37	
7.07–29.16	237	0.01 (−0.02, 0.04) 0.56		0.06 (0.01, 0.11) 0.01		0.02 (−0.01, 0.04) 0.24	
Lymphocyte count, ×10^9^/L							
0.1–0.95	224	0.01 (−0.02, 0.04) 0.67	0.818	0.06 (0.00, 0.11) 0.04	0.677	0.01 (−0.02, 0.04) 0.48	0.953
1–1.39	215	0.00 (−0.03, 0.04) 0.76		0.03 (−0.04, 0.11) 0.42		0.02 (−0.01, 0.04) 0.29	
1.4–4.5	270	0.02 (−0.01, 0.05) 0.27		0.02 (−0.03, 0.08) 0.42		0.02 (−0.01, 0.05) 0.22	
Monocyte count, ×10^9^/L							
0–0.39	208	0.01 (−0.02, 0.03) 0.59	0.662	0.01 (−0.05, 0.08) 0.71	0.480	0.01 (−0.02, 0.03) 0.67	0.351
0.4–0.49	138	−0.00 (−0.04, 0.03) 0.82		0.02 (−0.07, 0.12) 0.67		−0.01 (−0.04, 0.03) 0.75	
0.5–2.9	363	0.02 (−0.01, 0.05) 0.25		0.06 (0.01, 0.10) 0.03		0.03 (0.00, 0.05) 0.03	
Phosphorus, mmol/L							
0.41–1	235	0.00 (−0.03, 0.04) 0.80	0.494	0.03 (−0.02, 0.08) 0.30	1.000	0.02 (−0.01, 0.05) 0.24	0.450
1.01–1.15	222	0.03 (−0.01, 0.07) 0.16		0.03 (−0.03, 0.09) 0.38		0.03 (0.00, 0.07) 0.04	
1.16–2.31	252	0.00 (−0.03, 0.03) 0.96		0.03 (−0.06, 0.11) 0.52		−0.00 (−0.03, 0.02) 0.74	
Platelet count, ×10^9^/L							
10–142	230	0.00 (−0.03, 0.04) 0.86	0.543	0.06 (0.00, 0.12) 0.049	0.553	0.03 (−0.00, 0.06) 0.07	0.370
143–191	242	0.02 (−0.01, 0.05) 0.15		0.03 (−0.04, 0.09) 0.39		0.02 (−0.01, 0.04) 0.21	
192–515	237	−0.00 (−0.04, 0.03) 0.86		0.02 (−0.05, 0.08) 0.67		−0.00 (−0.04, 0.03) 0.78	

BMI, body mass index. Adjusted for age, BMI, hemoglobin, phosphorus, neutrophil, lymphocyte, platelet, and monocyte, except the subgroup variable.

## 4 Discussion

The present retrospective analysis study aimed to examine a correlation between diabetes status and trochanteric BMD in a diverse group of 710 admitted OFF patients. Various subgroup analyses were carried out to correspond with the identified variables, diabetes status, and trochanteric BMD, in addition to an examination of patient profiles. By incorporating further variables into the fully adjusted model, the findings reveal a positive and remarkable relationship between diabetes status and trochanteric BMD in patients with OPFs. The results indicated that patients with diabetes possessed substantially higher trochanteric BMD values than those without diabetes. Further, based on sex identity, male patients exhibited a significant positive relationship between their diabetes status and trochanteric BMD, whereas no such correlation was noted in female patients.

Multiple studies demonstrated a strong relationship between diabetes status and BMD. Different populations, including postmenopausal women (29), and elderly individuals (30, 31), have exhibited this correlation. A meta-analysis encompassing 15 studies with a total of 852,705 male and female participants revealed that individuals with diabetes mellitus exhibit significantly higher BMD compared to non-diabetic individuals. The analysis of individual participant data demonstrated a consistent association between type 2 diabetes and increased femoral neck BMD (FN-BMD) at baseline, with coefficients of *β* = 0.029 (95% CI 0.018–0.041) for males and *β* = 0.046 (95% CI 0.039–0.053) for females. After adjusting for BMI, although this association was attenuated, it remained statistically significant, with coefficients of *β* = 0.013 (95% CI 0.01–0.025) for males and *β* = 0.022 (95% CI 0.015–0.029) for females (32). These studies indicate that individuals with diabetes typically exhibit higher BMD compared to those without diabetes. Our study corroborates the findings of most previous studies, demonstrating that patients with type 2 diabetes exhibit higher trochanteric BMD compared to non-diabetic individuals. Similarly, this research contributes to the existing literature by highlighting significant sex differences, particularly the stronger correlation between diabetic status and increased BMD observed in male patients.

However, some studies have suggested that male veterans (33), young females (34), and elderly males (35) exhibit a negative relationship between diabetes status and BMD. In contrast to healthy controls of the same age and sex, people with type 2 diabetes possessed substantially lower BMD in the lumbar spine and femoral neck, based on the findings of a prospective cross-sectional study executed in India. Bone loss and osteoporosis are suggested as consequences of diabetes in the study (36). Moreover, some observational studies (37) have indicated that diabetes and the incidence of osteoporosis and fractures do not appear to have a causal connection. These discrepant findings may be attributed to heterogeneity in study methodologies, diagnostic criteria, population characteristics, and individual demographic factors. In our investigation, we observed a positive independent association between diabetes status and trochanteric BMD, specifically in patients with OPFs.

The precise mechanism by which diabetes is associated with bone metabolism is still not fully defined. Several interconnected pathways may explain the increased bone density observed in diabetic patients. Different studies have suggested that the anabolic effect of insulin on bone tissue has been linked to higher BMD (38). Insulin resistance, characterized by decreased cellular responsiveness to insulin signaling, is a hallmark of type 2 diabetes. Hyperinsulinemia is a compensatory response characterized by elevated circulating insulin levels due to pancreatic β-cell hypersecretion (39). Due to the anabolic impact of insulin on bone metabolism, people with hyperinsulinemia demonstrate an increased BMD (40). Further, the synthesis and regulation of sex hormone-binding globulin (SHBG) may be influenced by hyperinsulinemia. SHBG is a protein that binds to reproductive hormones, including testosterone and estrogen, to decrease their bioavailability. However, hyperinsulinemia has the potential to inhibit hepatic SHBG secretions, thereby leading to a decline in SHBG levels in circulation. This reduction in SHBG levels results in increased concentrations of free, biologically active reproductive hormones (41). Enhancement of these secreted hormones has been associated with favorable impacts on bone health (42, 43). Further research is necessary to determine the exact mechanisms that support these findings and to explore possible therapeutic options that target inflammation and bone health in diabetic patients.

The findings of this study revealed a substantial increase in trochanteric BMD in males with diabetes relative to control participants; however, no such correlation was observed in females. This innovative result contrasts with previous studies and emphasizes the need to consider sex-specific impacts when evaluating bone health and fracture risks in diabetic patients. The underlying mechanisms for this sex-specific variation in BMD among diabetic patients remain complex and multifaceted. This might be associated with the differential impact of sex hormones, particularly in postmenopausal women. Estrogen plays a crucial role in bone metabolism, primarily by inhibiting the activity of osteoclasts, which are cells responsible for bone resorption. Furthermore, estrogen binds to specific receptors on osteoclasts, suppressing their formation and activity, and promoting apoptosis (44). This hormonal interaction helps maintain bone density by balancing the rates of bone formation and resorption. However, estrogen levels are significantly reduced in postmenopausal women, substantially reducing its protective effect on bone mass. During the postmenopausal period, this reduction in estrogen level increases osteoclast activity and accelerates bone resorption, consequently decreasing bone density and increasing fracture risk (45). This compromised hormonal protection may explain why females with diabetes do not exhibit the increased BMD observed in their male counterparts.

Another potential mechanism for the increased BMD in diabetic patients relates to elevated levels of insulin and insulin-like growth factor-1 (IGF-1), particularly during the early stages of diabetes or in cases characterized by insulin resistance. These anabolic hormones promote bone formation and increase BMD (46). A cross-sectional study revealed sex-specific differences in the relationship between IGF-1 levels, BMD, and fracture risk among Chinese patients with T2DM. Moreover, in men, IGF-1 levels were positively correlated with BMD at the femoral neck and total hip, and negatively linked with the 10-year probability of major osteoporotic fractures (MOFs) and hip fractures (HFs) (47). These data further validate the potential role of IGF-1 in enhancing BMD. The observed sexual dimorphism in IGF-1’s effects on bone metabolism could be attributed to multiple factors, including sex-specific variations in hormonal profiles, body composition parameters (muscle mass and fat distribution), and fundamental differences in skeletal architecture.

Despite higher bone density, diabetes patients still indicate an elevated risk of fractures. This phenomenon, known as the “diabetic paradox of bone fragility,” suggests that factors other than bone density may also influence fracture risk (14). This paradoxical relationship challenges conventional assessments of fracture risk that primarily rely on bone mineral density measurements. A cohort study in Canada identified diabetes as a significant independent risk factor for severe osteoporotic fractures among individuals aged ≥40, with a hazard ratio of 1.32 (95% CI 1.20–1.46) (48). Moreover, alterations in bone microarchitecture and material properties may play a critical role. Therefore, the National Bone Health Alliance recommends using parameters such as trabecular microarchitecture or cortical porosity to diagnose osteoporosis in T2DM patients (49). Diabetes is also associated with increased advanced glycation end-products (AGEs) in the bone matrix, which may reduce bone toughness (50). The accumulation of AGEs affects collagen cross-linking and compromises the mechanical properties of bone tissue (51). Moreover, diabetic complications such as neuropathy could increase the risk of falls, which further elevates fracture risk independently of bone density (52). This multifactorial nature of fracture risk in diabetes necessitates a comprehensive approach to prevention and treatment.

The findings of this study carry significant clinical implications. Primarily, it underscores the importance of diabetes as a potential determinant of bone density in patients with osteoporosis, particularly in males. Currently, the diagnosis of osteoporosis is primarily based on bone density measurements (53). For diabetic patients, even those with higher bone density than non-diabetic individuals, treatment should be considered at more favorable bone density levels due to the potential underestimation of their fracture risk (54). Recently, the American Diabetes Association updated its 2024 Standards of Medical Care in Diabetes, suggesting that a *T*-score of −2.0 in diabetic patients should be interpreted as equivalent to a *T*-score of −2.5 in non-diabetic patients (55). Therefore, clinicians assessing fracture risk and designing treatment plans for diabetic patients should incorporate diabetes as a significant factor in their fracture risk evaluations. This approach may facilitate the identification of patients requiring targeted preventive interventions. It is crucial to note that higher bone density in diabetic patients does not necessarily correlate with a lower risk of fractures. As demonstrated by Schwartz et al. (56), diabetic patients exhibited higher fracture rates despite elevated BMD values. Due to the focus of this study on a population with osteoporotic fractures, a comprehensive assessment of fracture risk was not feasible. Future studies should address the fracture and recurrent fracture risks in this specific population. Similarly, these findings could significantly contribute to the development of treatment strategies for osteoporosis in diabetic patients. In particular, rigorous glycemic control may be especially crucial for patients prone to osteoporotic fractures. Further investigations are warranted to elucidate the underlying mechanisms and to investigate potential therapeutic targets to enhance bone health in this specific demographic.

Multiple significant strengths are evident in the present study. Initially, a comprehensive screening procedure was employed to select the study participants. Further, a variety of potential confounding variables have been adjusted in three distinct models that investigated the relationship between diabetes status and trochanteric BMD meticulously. Further, sensitivity analyses were carried out to verify the reliability and accuracy of the outcomes and to support the integrity of our findings.

However, there are limitations to this study. Firstly, due to restricted database information, we were unable to include several factors associated with fracture risk, including the duration of diabetes, its complications (notably neuropathy), the use of anti-diabetic medications, other treatments, patients’ history of falling, and body composition data such as muscle mass and fat mass. In addition, baseline *T*-score data was not collected. Although *T*-scores are crucial for assessing comparative bone density metrics, our study relied on data extracted from the electronic medical records of hospitalized patients. The current configuration of our electronic medical record system does not support the direct extraction of *T*-scores from DXA imaging reports into our research database. This technical constraint prevented the inclusion of these valuable metrics. Future studies should consider incorporating these indicators to more comprehensively elucidate their association with fracture risk. Furthermore, this study employed a retrospective analysis design. While we did observe a correlation between diabetes status and trochanteric BMD in the patients, this finding does not provide sufficient evidence to support a causal connection. To establish causality, specific treatment trials are required. Moreover, the study was carried out in a single center using a comparatively limited sample size. To address these limitations, conducting extensive, multicenter randomized controlled trials that comprise a wide range of racial and ethnic groups is crucial to improve the accuracy and validity of resultant outcomes. Future research directions should include a greater number of relevant indicators, longitudinal study designs, and larger and more diverse populations.

## 5 Conclusion

This study revealed novel insights demonstrating that diabetes is linked to elevated trochanteric BMD, specifically in male patients, which deviates from previous research findings. These findings highlight the value of adjusting for sex-specific effects and underscore the importance of further comprehensive investigations, thereby enhancing our knowledge of the correlation between diabetic status and bone health. These findings have important implications for the development of protective and treatment strategies for osteoporosis in diabetic patients.

## Data Availability

The raw data supporting the conclusions of this article will be made available by the authors, without undue reservation.

## Footnotes

^1^ http://www.empowerstats.com

^2^ http://www.R-project.org

## References

[1] Pignolo RJ Law SF Chandra A. Bone aging, cellular senescence, and osteoporosis. JBMR Plus. (2021) 5:e10488. doi: 10.1002/jbm4.10488, PMID: 33869998 PMC8046105

[2] AlYami A Alosaimi MN Alshehri MS Alghamdi AT SaemAldahar M Alsafrani TA. Association between osteoporosis and refracture rate among patients with hip fractures at King Abdulaziz Medical City, Saudi Arabia. Cureus. (2022) 14:e22171. doi: 10.7759/cureus.22171, PMID: 35308740 PMC8923245

[3] Liu YC Yang TI Huang SW Kuo YJ Chen YP. Associations of the neutrophil-to-lymphocyte ratio and platelet-to-lymphocyte ratio with osteoporosis: a meta-analysis. Diagnostics. (2022) 12:2968. doi: 10.3390/diagnostics12122968 36552975 PMC9776713

[4] Heilmann NZ Reeves KW Hankinson SE. Phthalates and bone mineral density: a systematic review. Environ Health. (2022) 21:108. doi: 10.1186/s12940-022-00920-5, PMID: 36369032 PMC9652984

[5] Lee WT Fang YW Chen M Liou HH Lee CJ Tsai MH. Serum intact fibroblast growth factor 23 levels are negatively associated with bone mineral density in chronic hemodialysis patients. J Clin Med. (2023) 12:1550. doi: 10.3390/jcm12041550 36836085 PMC9964480

[6] Huang CF Pan PJ Chiang YH Yang SH. A rehabilitation-based multidisciplinary care model reduces hip fracture mortality in older adults. J Multidiscip Healthc. (2021) 14:2741–7. doi: 10.2147/JMDH.S331136, PMID: 34616155 PMC8488040

[7] Adeyemi A Delhougne G. Incidence and economic burden of intertrochanteric fracture: a medicare claims database analysis. JB JS Open Access. (2019) 4:e0045. doi: 10.2106/JBJS.OA.18.00045, PMID: 31161153 PMC6510469

[8] Bovbjerg PE Larsen MS Madsen CF Schønnemann J. Failure of short versus long cephalomedullary nail after intertrochanteric fractures. J Orthop. (2020) 18:209–12. doi: 10.1016/j.jor.2019.10.018, PMID: 32055145 PMC7005477

[9] Cho NH Shaw JE Karuranga S Huang Y da Rocha Fernandes JD Ohlrogge AW. IDF diabetes atlas: global estimates of diabetes prevalence for 2017 and projections for 2045. Diabetes Res Clin Pract. (2018) 138:271–81. doi: 10.1016/j.diabres.2018.02.023 29496507

[10] Yang Y Lin Y Wang M Yuan K Wang Q Mu P. Targeting ferroptosis suppresses osteocyte glucolipotoxicity and alleviates diabetic osteoporosis. Bone Res. (2022) 10:26. doi: 10.1038/s41413-022-00198-w, PMID: 35260560 PMC8904790

[11] Poiana C Capatina C. Fracture risk assessment in patients with diabetes mellitus. J Clin Densitom. (2017) 20:432–43. doi: 10.1016/j.jocd.2017.06.011 28716499

[12] Bai J Gao Q Wang C Dai J. Diabetes mellitus and risk of low-energy fracture: a meta-analysis. Aging Clin Exp Res. (2020) 32:2173–86. doi: 10.1007/s40520-019-01417-x, PMID: 31768878

[13] Losada-Grande E Hawley S Soldevila B Martinez-Laguna D Nogues X Diez-Perez A. Insulin use and excess fracture risk in patients with type 2 diabetes: a propensity-matched cohort analysis. Sci Rep. (2017) 7:3781. doi: 10.1038/s41598-017-03748-z, PMID: 28630427 PMC5476619

[14] Romero-Díaz C Duarte-Montero D Gutiérrez-Romero SA Mendivil CO. Diabetes and bone fragility. Diabetes Ther. (2021) 12:71–86. doi: 10.1007/s13300-020-00964-1, PMID: 33185853 PMC7843783

[15] Wang H Ba Y Xing Q du JL. Diabetes mellitus and the risk of fractures at specific sites: a meta-analysis. BMJ Open. (2019) 9:e024067. doi: 10.1136/bmjopen-2018-024067, PMID: 30610024 PMC6326306

[16] Vilaca T Walsh J Eastell R. Discordant pattern of peripheral fractures in diabetes: a meta-analysis on the risk of wrist and ankle fractures. Osteoporos Int. (2019) 30:135–43. doi: 10.1007/s00198-018-4717-0, PMID: 30306223

[17] Khosla S Samakkarnthai P Monroe DG Farr JN. Update on the pathogenesis and treatment of skeletal fragility in type 2 diabetes mellitus. Nat Rev Endocrinol. (2021) 17:685–97. doi: 10.1038/s41574-021-00555-5, PMID: 34518671 PMC8605611

[18] Yoon SH Kim BR Lee SY Beom J Choi JH Lim JY. Influence of comorbidities on functional outcomes in patients with surgically treated fragility hip fractures: a retrospective cohort study. BMC Geriatr. (2021) 21:283. doi: 10.1186/s12877-021-02227-5, PMID: 33910513 PMC8082882

[19] Martiniakova M Biro R Penzes N Sarocka A Kovacova V Mondockova V. Links among obesity, type 2 diabetes mellitus, and osteoporosis: bone as a target. Int J Mol Sci. (2024) 25:4827. doi: 10.3390/ijms25094827, PMID: 38732046 PMC11084398

[20] Kurajoh M Inaba M Motoyama K Kuriyama N Ozaki E Koyama T. Inverse association of plasma leptin with cortical thickness at distal radius determined with a quantitative ultrasound device in patients with type 2 diabetes mellitus. J Diabetes Investig. (2020) 11:174–83. doi: 10.1111/jdi.13071, PMID: 31074113 PMC6944815

[21] Zhang Y Jiang J Shen H Chai Y Wei X Xie Y. Total flavonoids from Rhizoma Drynariae (Gusuibu) for treating osteoporotic fractures: implication in clinical practice. Drug Des Devel Ther. (2017) 11:1881–90. doi: 10.2147/DDDT.S139804, PMID: 28694688 PMC5491704

[22] Camacho PM Petak SM Binkley N Diab DL Eldeiry LS Farooki A. American Association of Clinical Endocrinologists/American College of Endocrinology Clinical Practice Guidelines for the diagnosis and treatment of postmenopausal osteoporosis-2020 update. Endocr Pract. (2020) 26:1–46. doi: 10.4158/GL-2020-0524SUPPL, PMID: 32427503

[23] Lu K Wu YM Shi Q Gong YQ Zhang T Li C. A novel fracture liaison service using digital health: impact on mortality in hospitalized elderly osteoporotic fracture patients. Osteoporos Int. (2024) 35:53–67. doi: 10.1007/s00198-023-06905-5, PMID: 37698600

[24] Koro CE Bowlin SJ Bourgeois N Fedder DO. Glycemic control from 1988 to 2000 among U.S. adults diagnosed with type 2 diabetes: a preliminary report. Diabetes Care. (2004) 27:17–20. doi: 10.2337/diacare.27.1.17 14693960

[25] Tian X Zuo Y Chen S Wu S Wang A Luo Y. High serum uric acid trajectories are associated with risk of myocardial infarction and all-cause mortality in general Chinese population. Arthritis Res Ther. (2022) 24:149. doi: 10.1186/s13075-022-02812-y, PMID: 35729670 PMC9210742

[26] Horvath B Kloesel B Todd MM Cole DJ Prielipp RC. The evolution, current value, and future of the American Society of Anesthesiologists Physical Status Classification System. Anesthesiology. (2021) 135:904–19. doi: 10.1097/ALN.0000000000003947 34491303

[27] Charlson ME Carrozzino D Guidi J Patierno C. Charlson comorbidity index: a critical review of clinimetric properties. Psychother Psychosom. (2022) 91:8–35. doi: 10.1159/000521288, PMID: 34991091

[28] Kernan WN Viscoli CM Brass LM Broderick JP Brott T Feldmann E. Phenylpropanolamine and the risk of hemorrhagic stroke. N Engl J Med. (2000) 343:1826–32. doi: 10.1056/NEJM200012213432501, PMID: 11117973

[29] Du YJ Liu NN Zhong X Pan TR. Risk factors for nonalcoholic fatty liver disease in postmenopausal women with type 2 diabetes mellitus and the correlation with bone mineral density at different locations. Diabetes Metab Syndr Obes. (2022) 15:1925–34. doi: 10.2147/DMSO.S364804, PMID: 35761888 PMC9233539

[30] Yuan J Jia P Zhou JB. Comparison of bone mineral density in US adults with diabetes, prediabetes and normoglycemia from 2005 to 2018. Front Endocrinol. (2022) 13:890053. doi: 10.3389/fendo.2022.890053, PMID: 35712240 PMC9195625

[31] Samelson EJ Demissie S Cupples LA Zhang X Xu H Liu CT. Diabetes and deficits in cortical bone density, microarchitecture, and bone size: Framingham HR-pQCT study. J Bone Miner Res. (2018) 33:54–62. doi: 10.1002/jbmr.3240, PMID: 28929525 PMC5771832

[32] Koromani F Oei L Shevroja E Trajanoska K Schoufour J Muka T. Vertebral fractures in individuals with type 2 diabetes: more than skeletal complications alone. Diabetes Care. (2020) 43:137–44. doi: 10.2337/dc19-0925, PMID: 31658976 PMC7411280

[33] Yaturu S Humphrey S Landry C Jain SK. Decreased bone mineral density in men with metabolic syndrome alone and with type 2 diabetes. Med Sci Monit. (2009) 15:CR5-9 PMID: 19114969

[34] Mastrandrea LD Wactawski-Wende J Donahue RP Hovey KM Clark A Quattrin T. Young women with type 1 diabetes have lower bone mineral density that persists over time. Diabetes Care. (2008) 31:1729–35. doi: 10.2337/dc07-2426 18591404 PMC2518333

[35] Wang X Pei Y. Correlation of bone mineral density with disease duration and body mass in elder men with type 2 diabetes mellitus. Chin J Tissue Eng Res. (2008) 15:2891–2894.

[36] Mathen PG Thabah MM Zachariah B das A. Decreased bone mineral density at the femoral neck and lumbar spine in South Indian patients with type 2 diabetes. J Clin Diagn Res. (2015) 9:OC08-12. doi: 10.7860/JCDR/2015/14390.6450, PMID: 26500934 PMC4606263

[37] Asokan AG Jaganathan J Philip R Soman RR Sebastian ST Pullishery F. Evaluation of bone mineral density among type 2 diabetes mellitus patients in South Karnataka. J Nat Sci Biol Med. (2017) 8:94–8. doi: 10.4103/0976-9668.198363, PMID: 28250682 PMC5320831

[38] An Y Zhang H Wang C Jiao F Xu H Wang X. Activation of ROS/MAPKs/NF-κB/NLRP3 and inhibition of efferocytosis in osteoclast-mediated diabetic osteoporosis. FASEB J. (2019) 33:12515–27. doi: 10.1096/fj.201802805RR, PMID: 31461386 PMC6902677

[39] Ahn J Baik JW Kim D Choi K Lee S Park SM. *In vivo* photoacoustic monitoring of vasoconstriction induced by acute hyperglycemia. Photoacoustics. (2023) 30:100485. doi: 10.1016/j.pacs.2023.100485, PMID: 37082618 PMC10112177

[40] Yoo KO Kim MJ Ly SY. Association between vitamin D intake and bone mineral density in Koreans aged ≥ 50 years: analysis of the 2009 Korea National Health and Nutrition Examination Survey using a newly established vitamin D database. Nutr Res Pract. (2019) 13:115–25. doi: 10.4162/nrp.2019.13.2.115, PMID: 30984355 PMC6449542

[41] Yang PJ Hou MF Ou-Yang F Tsai EM Wang TN. Association of early-onset breast cancer with body mass index, menarche, and menopause in Taiwan. BMC Cancer. (2022) 22:259. doi: 10.1186/s12885-022-09361-2, PMID: 35277131 PMC8917681

[42] Wankhede S Mohan V Thakurdesai P. Beneficial effects of fenugreek glycoside supplementation in male subjects during resistance training: a randomized controlled pilot study. J Sport Health Sci. (2016) 5:176–82. doi: 10.1016/j.jshs.2014.09.005, PMID: 30356905 PMC6191980

[43] Mills EG Yang L Nielsen MF Kassem M Dhillo WS Comninos AN. The relationship between bone and reproductive hormones beyond estrogens and androgens. Endocr Rev. (2021) 42:691–719. doi: 10.1210/endrev/bnab015, PMID: 33901271 PMC8599211

[44] N'deh K Yoo HS Chung KH Lee KJ Kim DH Yoon JA. Collagen extract derived from Yeonsan Ogye chicken increases bone microarchitecture by suppressing the RANKL/OPG ratio via the JNK signaling pathway. Nutrients. (2020) 12:1967. doi: 10.3390/nu12071967, PMID: 32630655 PMC7400104

[45] Neri AA Galanis D Galanos A Pepe AE Soultanis K Zervas A. The effect of *Ceratonia siliqua* supplement on bone mineral density in ovariectomy-induced osteoporosis in rats. In Vivo. (2023) 37:270–85. doi: 10.21873/invivo.13077, PMID: 36593044 PMC9843798

[46] Saeki C Oikawa T Ueda K Nakano M Torisu Y Saruta M. Serum insulin-like growth factor 1 levels, facture risk assessment tool scores and bone disorders in patients with primary biliary cholangitis. Diagnostics. (2022) 12:1957. doi: 10.3390/diagnostics12081957 36010307 PMC9407172

[47] Lv F Cai X Zhang R Zhou L Zhou X Han X. Sex-specific associations of serum insulin-like growth factor-1 with bone density and risk of fractures in Chinese patients with type 2 diabetes. Osteoporos Int. (2021) 32:1165–73. doi: 10.1007/s00198-020-05790-6, PMID: 33415372

[48] Leslie WD Morin SN Lix LM Majumdar SR. Does diabetes modify the effect of FRAX risk factors for predicting major osteoporotic and hip fracture? Osteoporos Int. (2014) 25:2817–24. doi: 10.1007/s00198-014-2822-2, PMID: 25092059

[49] Siris ES Adler R Bilezikian J Bolognese M Dawson-Hughes B Favus MJ. The clinical diagnosis of osteoporosis: a position statement from the National Bone Health Alliance Working Group. Osteoporos Int. (2014) 25:1439–43. doi: 10.1007/s00198-014-2655-z, PMID: 24577348 PMC3988515

[50] Jaworski M Wierzbicka E Czekuć-Kryśkiewicz E Płudowski P Kobylińska M Szalecki M. Bone density, geometry, and mass by peripheral quantitative computed tomography and bone turnover markers in children with diabetes mellitus type 1. J Diabetes Res. (2022) 2022:1–16. doi: 10.1155/2022/9261512 PMC9038424 35480630

[51] Li S Li Y Xu X Shao J Xie R Liu S. Lens autofluorescence based advanced glycation end products (AGEs) measurement to assess risk of osteopenia among individuals under the age of 50. Med Devices. (2022) 15:341–7. doi: 10.2147/MDER.S381115, PMID: 36105561 PMC9467441

[52] Komorita Y Minami M Maeda Y Yoshioka R Ohkuma T Kitazono T. Prevalence of bone fracture and its association with severe hypoglycemia in Japanese patients with type 1 diabetes. BMJ Open Diabetes Res Care. (2021) 9:e002099. doi: 10.1136/bmjdrc-2020-002099, PMID: 33888545 PMC8070870

[53] Wang C Zhang Z Zheng Z Chen X Zhang Y Li C. Relationship between obstructive sleep apnea-hypopnea syndrome and osteoporosis adults: a systematic review and meta-analysis. Front Endocrinol. (2022) 13:1013771. doi: 10.3389/fendo.2022.1013771, PMID: 36465605 PMC9712780

[54] Ferrari SL Abrahamsen B Napoli N Akesson K Chandran M Eastell R. Diagnosis and management of bone fragility in diabetes: an emerging challenge. Osteoporos Int. (2018) 29:2585–96. doi: 10.1007/s00198-018-4650-2, PMID: 30066131 PMC6267152

[55] ElSayed NA Aleppo G Aroda VR Bannuru RR Brown FM Bruemmer D. 4. Comprehensive medical evaluation and assessment of comorbidities: standards of care in diabetes-2023. Diabetes Care. (2023) 46:S49–67. doi: 10.2337/dc23-S004, PMID: 36507651 PMC9810472

[56] Schwartz AV Vittinghoff E Bauer DC Hillier TA Strotmeyer ES Ensrud KE. Association of BMD and FRAX score with risk of fracture in older adults with type 2 diabetes. JAMA. (2011) 305:2184–92. doi: 10.1001/jama.2011.715, PMID: 21632482 PMC3287389

