# Supplementary material for: Association between diabetes mellitus and trochanteric bone mineral density in individuals with osteoporotic fractures: a retrospective study
Source: Front Med (Lausanne). 2024 Dec 17;11:1492603. doi: 10.3389/fmed.2024.1492603 (PMC11685145; doi:10.3389/fmed.2024.1492603)
Supplement: Supplementary file 1 [file Table_1.docx]

**Table S1** Patient characteristics based on different diabetes status

| **Characteristics** | **Non-Diabetes** | **Diabetes** | **P-value^a^** | **P-value^b^** |
| --- | --- | --- | --- | --- |
| N | 537 | 173 |  |  |
| Trochanteric BMD, mean ± SD, g/cm^2^ | 0.51 ± 0.10 | 0.52 ± 0.10 | 0.188 | 0.171 |
| Age, mean ± SD, years | 71.43 ± 10.23 | 71.28 ± 9.96 | 0.862 | 0.698 |
| BMI, mean ± SD, kg/m^2^ | 22.76 ± 3.26 | 22.34 ± 2.94 | 0.128 | 0.116 |
| Magnesium, mean ± SD, mmol/L | 0.89 ± 0.10 | 0.87 ± 0.10 | 0.002 | 0.011 |
| Sodium, mean ± SD, mmol/L | 141.33 ± 2.86 | 140.38 ± 2.62 | <0.001 | <0.001 |
| Phosphorus, mean ± SD, mmol/L | 1.10 ± 0.21 | 1.06 ± 0.26 | 0.105 | 0.036 |
| Platelet count, mean ± SD, ×10^9^/L | 175.60 ± 63.82 | 161.93 ± 52.83 | 0.011 | 0.012 |
| Hemoglobin, mean ± SD, g/L | 126.46 ± 18.38 | 120.57 ± 18.76 | <0.001 | <0.001 |
| Albumin, mean ± SD, g/L | 39.99 ± 4.06 | 39.52 ± 4.45 | 0.192 | 0.307 |
| Calcium, mean ± SD, mmol/L | 2.20 ± 0.13 | 2.20 ± 0.14 | 0.799 | 0.772 |
| Neutrophil count, mean ± SD, ×10^9^/L | 6.15 ± 2.87 | 7.38 ± 3.74 | <0.001 | <0.001 |
| Lymphocyte count, mean ± SD, ×10^9^/L | 1.32 ± 0.57 | 1.10 ± 0.53 | <0.001 | <0.001 |
| Monocyte count, mean ± SD, ×10^9^/L | 0.51 ± 0.26 | 0.44 ± 0.22 | 0.002 | 0.001 |
| ALT, mean ± SD, U/L | 23.17 ± 19.16 | 24.10 ± 36.08 | 0.662 | 0.558 |
| AST, mean ± SD, U/L | 24.97 ± 16.22 | 29.91 ± 62.96 | 0.098 | 0.696 |
| Cr, mean ± SD, μmol/L | 67.30 ± 28.97 | 71.98 ± 41.58 | 0.100 | 0.886 |
| BUN, mean ± SD, mmol/L | 5.97 ± 3.10 | 6.74 ± 3.07 | 0.005 | 0.007 |
| SUA, mean ± SD, μmol/L | 283.98 ± 98.93 | 284.85 ± 95.38 | 0.920 | 0.741 |
| HbA1c, mean ± SD, % | 5.93 ± 0.55 | 7.57 ± 1.85 | 0.016 | 0.003 |
| Sex, N (%) |  |  | 0.953 | - |
| Female | 414 (77.09%) | 133 (76.88%) |  |  |
| Male | 123 (22.91%) | 40 (23.12%) |  |  |
| Hypertension, N (%) |  |  | <0.001 | - |
| No | 458 (85.29%) | 122 (70.52%) |  |  |
| Yes | 79 (14.71%) | 51 (29.48%) |  |  |
| ASA score, N (%) |  |  | 0.130 | - |
| 1 | 37 (6.89%) | 12 (6.94%) |  |  |
| 2 | 367 (68.34%) | 107 (61.85%) |  |  |
| ≥3 | 133 (24.77%) | 54 (31.21%) |  |  |
| CCI score, N (%) |  |  | <0.001 | - |
| 0 | 502 (93.48%) | 125 (72.25%) |  |  |
| 1 | 26 (4.84%) | 38 (21.97%) |  |  |
| ≥2 | 9 (1.68%) | 10 (5.78%) |  |  |
| Fracture category, N (%) |  |  | 0.475 | - |
| Thoracic vertebra | 109 (20.30%) | 40 (23.12%) |  |  |
| Lumbar vertebra | 195 (36.31%) | 58 (33.53%) |  |  |
| Wrist | 15 (2.79%) | 7 (4.05%) |  |  |
| Proximal humerus | 40 (7.45%) | 11 (6.36%) |  |  |
| Femoral neck | 116 (21.60%) | 30 (17.34%) |  |  |
| Femoral trochanteric/subtrochanteric | 62 (11.55%) | 27 (15.61%) |  |  |

Abbreviations: SD, standard deviation; BMD, bone mineral density; BMI, body mass index; ALT, alanine aminotransferase; AST, aspartate aminotransferase; Cr, creatinine; BUN, blood urea nitrogen; SUA, serum uric acid; HbA1c, glycated hemoglobin; ASA, American Society of Anesthesiologists; CCI, Charlson comorbidity index.

^a^P-value: t-tests for continuous variables, chi-square tests for categorical variables.

^b^P-value: Kruskal Wallis rank test for continuous variables, Fisher exact for categorical variables with expects < 10.
