# Supplementary material for: Association between diabetes mellitus and trochanteric bone mineral density in individuals with osteoporotic fractures: a retrospective study
Source: Front Med (Lausanne). 2024 Dec 17;11:1492603. doi: 10.3389/fmed.2024.1492603 (PMC11685145; doi:10.3389/fmed.2024.1492603)
Supplement: Supplementary file 2 [file Table_2.docx]

**Table S2** Univariate analyses of factors associated with trochanteric BMD

| Variables | Statistics | β (95% CI) P-value |
| --- | --- | --- |
| Age, mean ± SD, years | 71.394 ± 10.159 | -0.004 (-0.004, -0.003) <0.001 |
| BMI, mean ± SD, kg/m^2^ | 22.656 ± 3.189 | 0.008 (0.006, 0.010) <0.001 |
| Magnesium, mean ± SD, mmol/L | 0.886 ± 0.098 | 0.047 (-0.029, 0.123) 0.226 |
| Sodium, mean ± SD, mmol/L | 141.096 ± 2.830 | -0.002 (-0.004, 0.001) 0.170 |
| Phosphorus, mean ± SD, mmol/L | 1.088 ± 0.224 | -0.056 (-0.089, -0.023) 0.001 |
| Platelet count, mean ± SD, ×10^9^/L | 172.263 ± 61.567 | -0.000 (-0.000, -0.000) 0.011 |
| Hemoglobin, mean ± SD, g/L | 125.027 ± 18.631 | 0.000 (-0.000, 0.000) 0.962 |
| Albumin, mean ± SD, g/L | 39.873 ± 4.158 | 0.001 (-0.001, 0.002) 0.516 |
| Calcium, mean ± SD, mmol/L | 2.202 ± 0.130 | -0.023 (-0.080, 0.035) 0.441 |
| Neutrophil count, mean ± SD, ×10^9^/L | 6.452 ± 3.144 | -0.001 (-0.003, 0.001) 0.459 |
| Lymphocyte count, mean ± SD, ×10^9^/L | 1.269 ± 0.572 | -0.012 (-0.025, 0.001) 0.068 |
| Monocyte count, mean ± SD, ×10^9^/L | 0.489 ± 0.256 | 0.013 (-0.016, 0.043) 0.367 |
| ALT, mean ± SD, U/L | 23.393 ± 24.360 | 0.000 (-0.000, 0.000) 0.952 |
| AST, mean ± SD, U/L | 26.170 ± 34.131 | -0.000 (-0.000, 0.000) 0.771 |
| Cr, mean ± SD, μmol/L | 68.439 ± 32.525 | -0.000 (-0.000, 0.000) 0.058 |
| BUN, mean ± SD, mmol/L | 6.157 ± 3.112 | -0.001 (-0.003, 0.001) 0.434 |
| SUA, mean ± SD, μmol/L | 284.194 ± 98.013 | -0.000 (-0.000, -0.000) 0.004 |
| HbA1c, mean ± SD, % | 7.046 ± 1.724 | 0.014 (-0.012, 0.040) 0.293 |
| Sex, N (%) |  |  |
| Female | 547 (77.042%) | Reference |
| Male | 163 (22.958%) | 0.071 (0.054, 0.088) <0.001 |
| Hypertension, N (%) |  |  |
| No | 580 (81.690%) | Reference |
| Yes | 130 (18.310%) | -0.009 (-0.028, 0.010) 0.358 |
| ASA score, N (%) |  |  |
| 1 | 49 (6.901%) | Reference |
| 2 | 474 (66.761%) | -0.039 (-0.068, -0.010) 0.010 |
| ≥3 | 187 (26.338%) | -0.077 (-0.108, -0.045) <0.001 |
| CCI score, N (%) |  |  |
| 0 | 627 (88.310%) | Reference |
| 1 | 64 (9.014%) | -0.003 (-0.029, 0.024) 0.845 |
| ≥2 | 19 (2.676%) | -0.012 (-0.059, 0.034) 0.606 |
| Fracture category, N (%) |  |  |
| Thoracic vertebra | 149 (20.986%) | Reference |
| Lumbar vertebra | 253 (35.634%) | 0.028 (0.008, 0.049) 0.007 |
| Wrist | 22 (3.099%) | 0.025 (-0.020, 0.070) 0.282 |
| Proximal humerus | 51 (7.183%) | 0.016 (-0.016, 0.048) 0.333 |
| Femoral neck | 146 (20.563%) | -0.011 (-0.034, 0.012) 0.364 |
| Femoral trochanteric/subtrochanteric | 89 (12.535%) | -0.001 (-0.028, 0.025) 0.915 |

Abbreviations: SD, standard deviation; Q1, first quartile; Q3, third quartile; BMD, bone mineral density; BMI, body mass index; ALT, alanine aminotransferase; AST, aspartate aminotransferase; Cr, creatinine; BUN, blood urea nitrogen; SUA, serum uric acid; HbA1c, glycated hemoglobin; ASA, American Society of Anesthesiologists; CCI, Charlson comorbidity index.
