# Supplementary material for: Association between diabetes mellitus and trochanteric bone mineral density in individuals with osteoporotic fractures: a retrospective study
Source: Front Med (Lausanne). 2024 Dec 17;11:1492603. doi: 10.3389/fmed.2024.1492603 (PMC11685145; doi:10.3389/fmed.2024.1492603)
Supplement: Supplementary file 3 [file Table_3.docx]

**Table S3** Association between Diabetes Status and BMD at Different regions in the Fully Adjusted Model

|  | **Diabetes** | **Female** | **Male** | **Total** |
| --- | --- | --- | --- | --- |
| **Primary outcome** |  |  |  |  |
| Trochanteric BMD | No | Reference | Reference | Reference |
|  | Yes | 0.007 (-0.011, 0.025) 0.463 | 0.039 (0.004, 0.074) 0.029 | 0.015 (-0.001, 0.031) 0.069 |
| **Secondary outcomes** |  |  |  |  |
| Lumbar spine BMD | No | Reference | Reference | Reference |
|  | Yes | 0.024 (-0.002, 0.051) 0.073 | 0.026 (-0.031, 0.083) 0.373 | 0.026 (0.002, 0.050) 0.036 |
| Femoral neck BMD | No | Reference | Reference | Reference |
|  | Yes | 0.011 (-0.022, 0.044) 0.521 | 0.006 (-0.058, 0.069) 0.857 | 0.012 (-0.017, 0.041) 0.404 |
| Ward's triangle BMD | No | Reference | Reference | Reference |
|  | Yes | 0.014 (-0.011, 0.040) 0.282 | -0.013 (-0.071, 0.044) 0.647 | 0.009 (-0.015, 0.032) 0.466 |
| Overall average BMD | No | Reference | Reference | Reference |
|  | Yes | 0.007 (-0.016, 0.030) 0.556 | 0.014 (-0.033, 0.061) 0.557 | 0.011 (-0.010, 0.032) 0.311 |

Data in the table: β (95%CI) *p*-value.

Adjusted for age, BMI, hemoglobin, neutrophil, lymphocyte, monocyte, phosphorus and platelet.
