# Supplementary material for: Association between diabetes mellitus and trochanteric bone mineral density in individuals with osteoporotic fractures: a retrospective study
Source: Front Med (Lausanne). 2024 Dec 17;11:1492603. doi: 10.3389/fmed.2024.1492603 (PMC11685145; doi:10.3389/fmed.2024.1492603)
Supplement: Supplementary file 4 [file Table_4.docx]

**Table S4** Association between Diabetes status and Trochanteric BMD with Different Adjusted Variables

|  | Diabetes | Female | Male | Total |
| --- | --- | --- | --- | --- |
| Model 1 | No | Reference | Reference | Reference |
|  | Yes | 0.010 (-0.008, 0.028) 0.256 | 0.040 (0.006, 0.075) 0.022 | 0.017 (0.001, 0.033) 0.035 |
| Model 2 | No | Reference | Reference | Reference |
|  | Yes | 0.005 (-0.012, 0.022) 0.576 | 0.037 (0.003, 0.070) 0.035 | 0.012 (-0.003, 0.027) 0.123 |
| Model 3 | No | Reference | Reference | Reference |
|  | Yes | 0.005 (-0.013, 0.023) 0.579 | 0.049 (0.011, 0.087) 0.013 | 0.013 (-0.003, 0.030) 0.108 |

Data in the table: β (95%CI) *p*-value, Outcome variable: Trochanteric BMD, Exposed variables: Diabetes.

Model 1 adjusted for age, BMI, hemoglobin, neutrophil, lymphocyte, monocyte, phosphorus and platelet.

Model 2 adjusted for age, BMI, lymphocyte, monocyte, phosphorus and platelet.

Model 3 adjusted for age, BMI, lymphocyte, monocyte, phosphorus, platelet, BUN, Hypertension, ASA score and CCI score.

Abbreviations: BMD, bone mineral density; BMI, body mass index; BUN, blood urea nitrogen; ASA, American Society of Anesthesiologists; CCI, Charlson comorbidity index.
